# Peridynamic Simulation to Fracture Mechanism of CBN Grain in the Honing Wheel Dressing Process

**DOI:** 10.3390/mi12101186

**Published:** 2021-09-29

**Authors:** Fuwei Wang, Yuanlong Chen, Yang Gao, Yuan Liang, Jie Su, Lin Liu

**Affiliations:** 1 School of Mechanical Engineering, Hefei University of Technology, Hefei 230009, China; nuaawfw@163.com (F.W.); 2008034@nmu.edu.cn (Y.G.); 2 College of Mechatronic Engineering, North Minzu University, Yinchuan 750021, China; ly18895087502@163.com; 3 School of Mechanical Engineering, Ningxia University, Yinchuan 750021, China; sujie9493@163.com; 4 Department of Mechanical Engineering, University of Kansas, Lawrence, KS 66045, USA; linliu@ku.edu

**Keywords:** bond-based peridynamics (PD), CBN grain, dressing, honing wheel, grain fracture

## Abstract

Regularly dressing of CBN honing wheel is an effective way to keep its sharpness and correct geometry during honing process. This study aims to understand the fracture mechanism of single CBN grain in the dressing process of honing wheel. The honing wheel dressing process was simplified into the dressing process of grinding wheel, and the bond-based Peridynamic method considering bond rotation effect was developed to investigate the progressive fracture evolution, stress characteristics, and fracture modes of CBN grains in this process. It was found that fracture evolution of CBN grains mainly underwent four stages: elastic deformation, damage initiation, crack formation, and macro fracture. In addition, the fracture initiation and propagation were mainly determined by the tensile and shear stress, where the former led to mode I fractures and the latter led to mode II fractures. The propagation of mode I fractures was stable while the propagation of mode II fracture was unstable. The results show that the Peridynamic approach has great potential to predict the fracture mechanism of CBN grain in the dressing process of honing and grinding wheels.

## 1. Introduction

Gear honing is a cost-efficient process for the manufacturing of gears due to its low noise and beneficial wear characteristics [1]. Therefore, it is usually used in the precision machining of gears. In gear honing process, the abrasive grains on the tooth surface of honing wheel will wear. The worn abrasive grains are likely to cause uneven wear on the surface of the honing wheel, and the resulting contour error of the honing wheel will be directly copied to the surface of the workpiece, resulting in a decrease in the surface roughness and surface accuracy of the workpiece [2]. In order to keep the surface of the honing wheel with sufficient sharpness and correct geometry during the honing process, it must be regularly dressed [3]. As a superhard material with excellent thermal and chemical stability, cubic boron nitride (CBN) grain is widely used in the grinding and honing of gears [4,5,6]. The existing research on dressing of CBN grain mainly focused on grinding, and the research on the dressing of honing wheel in gear honing process is seldom reported. Gear honing process is the interaction between the honing wheel and workpiece gear, which can be equivalent to a grinding process by introducing the effective diameters of honing wheel and workpiece gear [7]. Hence, the dressing mechanism of abrasive grains on honing wheel in gear honing process can be understood by means of the grinding process.

The dressing of CBN grinding wheel has always had problems such as long time, low precision and large tool loss, which seriously hinders the further promotion and application of such high-performance, multi-purpose grinding wheels [8]. Therefore, it has been an important research topic in the manufacturing field about how to achieve high-efficiency, high-precision and high-quality dressing of super-abrasive grinding wheels [2]. To achieve this goal, the most common methods used by researchers in recent years include mechanical dressing (MD) method [9,10,11], electrical discharge dressing (EDD) method [12,13,14], electrolytic in-process dressing (ELID) method [15] and Pulsed laser dressing (PLD) method [16]. However, the dressing mechanics, specifically the grain fracture evolution mechanism and brittle fracture mechanics, have not been addressed in a detailed fashion, which further restrict people’s understanding about the influence of grain fracture on the accuracy, quality and efficiency of dressing [17].

Numerical simulation is an effective way to obtain the dynamic fracture process, which can obtain the instantaneous fracture state and is an important supplement to experimental study. The existing numerical simulation methods of abrasive fracture mainly include the finite element method (FEM) [18,19], molecular dynamics method (MD) [20,21], and the coupling approach of FEM and smooth particle hydrodynamics method (SPH) [22]. The MD method is capable of simulating the surface generation [23], abrasive wear [21] and surface roughness [24] in nanoscale machining processes, but it has limitation in dealing with micron to macro scale problems due to its high demand of computational resources. FEM is based on continuum mechanics to establish the equations of motion and constitutive models of materials. It has good advantages in solving the problems of continuum deformation. However, when solving the discontinuous problems such as crack propagation and breakage, etc., the damage and fracture criteria need to be introduced additionally due to the limitation of solving higher-order partial differential equations. The coupling of FEM and SPH can make up for the shortcomings of the above methods to some extent, but when dealing with nonlocal deformation, the SPH method suffers from “tension instability” [22].

Peridynamic (PD) theory can overcome the problems above. As a newly developed theory of nonlocal solid mechanics, PD allows any finite discontinuity in the displacement field and internal force field and does not need to introduce additional damage criteria to deal with discontinuous deformation. Therefore, it has been widely used to study the damage and fracture of various materials [25]. Silling proposed a bond-based PD model (PMB) to simulate the crack growth and damage of brittle materials under tensile and impact loads [26]. Subsequently, a various bond-based PD models have been developed and used to study the crack growth rule of brittle materials such as limestone [27], soda-lime glass [28] and concrete structures [29]. They verify that it is effective using the bond-based PD model to deal with discontinuous deformation.

However, the above bond-based PD theory only concerns the axial tension of bonds between the material points, so it’s Poisson’s ratio of material is fixed at 0.25. In order to eliminate this limitation, Zhu [30] and Zhou [31] developed the bond-based PD models considering the rotation effect or tangential effect of material bonds, which overcome the problem of fixed Poisson’s ratio of PMB model, and can effectively simulate the shear deformation behavior of materials. Hence, this model will be used to analyze the fracture of CBN grains in the present research.

This paper aims at describing the fracture mechanism of CBN grains in the honing wheel dressing process based on a revised bond-based PD model considering the bond rotation effect. By establishing the PD model of single CBN grain, we simulate the fracture behavior of three different CBN grains, and the fracture evolution, stress characteristics and fracture modes are investigated. The effectiveness of this method is verified by comparison the present approaches with experimental results and the existing experimental results.

## 2. PD Theory

### 2.1. Bond-Based PD Theory

**Figure 1:**
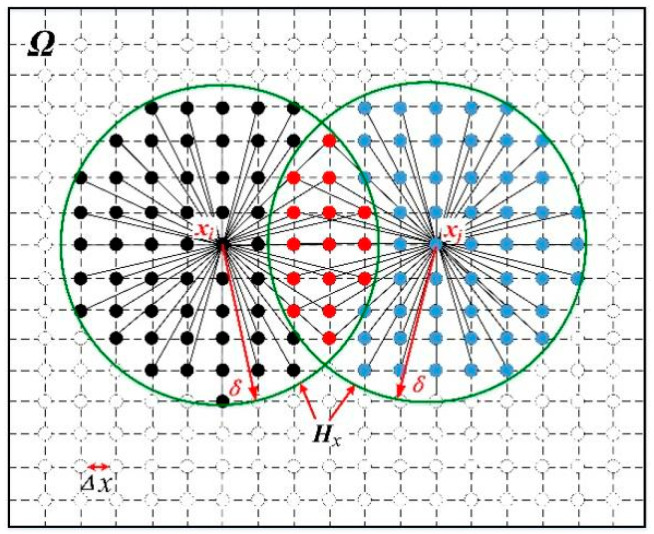
Each material point x interacts with points in the spherical domain $H_{x}$.

PD is a nonlocal theory. Its motion equation is established based on the integral of internal force function. Relative to the reference configuration as shown in Figure 1, the motion equation of the material point x at any time t is given as follows [25]

(1) $$\rho \left(x\right)\ddot{u}\left(x,t\right)\;=\int_{H_{x}}^{}f\left(x,x^{\prime },t\right)dV_{x^{\prime }}+b\left(x,t\right)$$

where $\rho \left(x\right)$ is mass density, $u\left(x,t\right)$ is the displacement field, $f\left(x,x^{\prime },t\right)$ is PD bond force between material points x and $x^{\prime }$, $b\left(x,t\right)$ is external body force, and $H_{x}$ a PD family at point x in a spatial domain Ω ($H_{x}\subset \Omega ,x\subset H_{x})$ determined by PD horizon δ. The bond force has the dimension of $force\cdot volume^{-2}$ and can be represented as

(2) $$f\left(x,x^{\prime },t\right)\;=f\left(\eta ,\xi \right)\;=f\left(\eta ,\xi \right)\;\cdot \;n,\;n=\frac{\eta +\xi }{\left|\eta +\xi \right|},\forall \eta ,\xi$$

where f is the scalar function of bond force f, n is the direction vector of material bond, $\eta =u^{\prime }-u$ is the relative displacement vector between material points, ξ is the relative position vector between material points, and η+ξ denotes the relative position vector between material points in the current configuration.

Silling [26] proposed a PMB (prototype micro elastic brittle) model in 2005 characterizing the constitutive behavior of micro elastic brittle materials. Assuming that the material is linear elastic and isotropic, its bond force function can be expressed as

(3) $$f\left(\eta ,\xi \right)\;=cs\left(\eta ,\xi \right),\;\forall \eta ,\xi$$

where c is the material bond constant and s the bond stretch ratio, which read

(4) $$c=\frac{18K}{\pi \delta^{4}},s\left(\eta ,\xi \right)\;=\frac{\left|\eta +\xi \right|-\left|\xi \right|}{\left|\xi \right|}\;$$

where K is the bulk modulus of the material.

### 2.2. Bond-Based PD Model with Rotation Effect

CBN grain can be regarded as a brittle material. In the actual cutting process, the deformation of abrasive grains often includes both tension/compression deformation and shear deformation. The PMB model can well describe the tension and compression deformation, but fails to characterize the shear deformation of materials. To formulate the tension/compression deformation and shear deformation of the material, it is necessary to introduce the rotation term of the material bond in the PMB model.

**Figure 2:**
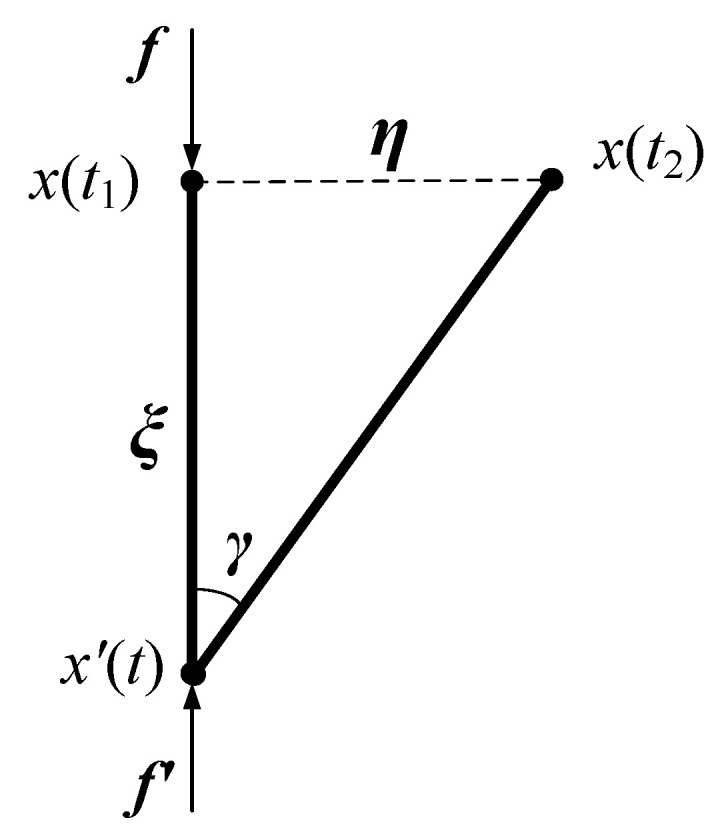
Rotation effect of PD bonds.

When considering the shear deformation of the PD bonds, as shown in Figure 2, the shear strain vector can be represented by the relative position vector ξ and the relative displacement vector η [30]

(5) $$\gamma =\frac{\eta -s\left(\eta ,\xi \right)\left|\xi \right|n}{\left|\xi \right|},\;s\left(\eta ,\xi \right)\;=\frac{\eta }{\left|\xi \right|}\cdot n\;$$

The micro-potential energy density for the micro linear elasticity can be formulated as [30]

(6) $$w_{\xi }=\frac{1}{2}cs^{2}\left|\xi \right|+\frac{1}{2}\kappa \gamma \cdot \gamma \left|\xi \right|\;=\frac{1}{2}\eta \cdot C\cdot \eta \;$$

where κ denotes the stiffness in the tangential direction, and

(7) $$C=\frac{1}{\left|\xi \right|}\left[c(n⊗n)\right]\;+\;\kappa \left(I-n⊗n\right)]\;$$

By combining Equations (6) and (7), the constitutive bond force (2) reads [30]

(8) $$f\left(\eta ,\xi \right)\;=\frac{\partial w}{\partial \eta }=cs\cdot n+\kappa \gamma$$

where c and κ represent the axial and tangential material parameters of PD bond respectively

(9) $$c=\frac{6E}{\pi \delta^{4}\left(1-2v\right)},\;\kappa =\frac{6E\left(1-4v\right)}{\pi \delta^{4}\left(1+v\right)\left(1-2v\right)}\;$$

where E is the Young’s modulus and v the Poisson’s ratio. When the rotation effect is neglected, κ=0, and Poisson’s ratio v=1/4, the model becomes the classical PMB model.

### 2.3. Damage Model

In PD theory, the deformation of the material bond has a limit. When the deformation exceeds this limit, the bond will break and cause the material to damage. Zhou [31] proposed a damage model based on bond potential energy and set $w_{c}$ as the critical micro-potential energy density. In fracture mechanics, the energy release rate $G_{c}$ is used to describe the critical energy of fracture. The relationship between $G_{c}$ and $w_{c}$ can be expressed as [31]

(10) $$G_{c}=\int_{0}^{\delta }\int_{0}^{2\pi }\int_{z}^{\delta }\int_{0}^{cos^{-1}z/\xi }w_{c}\delta^{2}sin\phi d\phi d\xi d\theta dz\;$$

By solving the integration, we can obtain the critical micro-potential energy density

(11) $$w_{c}=\frac{4G_{c}}{\pi \delta^{4}}$$

In the numerical simulation, the material micro-potential energy density $w_{\xi }$ in Equation (6) needs comparison with the critical micro-potential energy density at each step. When $w_{\xi }$ exceeds $w_{c}$, the material bond ξ breaks. The breakage of material bonds can be expressed by a historical dependent scalar function $\mu \left(w_{\xi },t\right)$ [31]

(12) $$\mu \left(w_{\xi },t\right)\;=\;\left\{\begin{array}{c} 1,0\leq w_{\xi }<w_{c}\\ 0,otherwise \end{array}\right.$$

The local damage at point x can be defined as the ratio of the number of broken bonds to the total number of bonds that interact directly with x

(13) $$D\left(x,t\right)\;=1-\frac{\int_{H_{x}}^{}\mu \left(w_{\xi },t\right)dV_{\xi }}{\int_{H_{x}}^{}dV_{\xi }}$$

The above equation shows that $0\leq D\left(x,t\right)\;\leq 1$. When $D\left(x,t\right)\;=1$, all the material bonds interacting with material point x will break; when $0<D\left(x,t\right)\;<1$, part of the bonds will break; when $D\left(x,t\right)\;=0$, no bond breaks.

### 2.4. Numerical Discretization

In the reference configuration, the object is discrete into material points with a certain volume. After discretization, the equation of motion (1) can be expressed as

(14) $$\rho {\ddot{u}}_{i}^{n}=\underset{p}{\sum }f(u_{p}^{n}-u_{i}^{n},x_{p}^{n}-x_{i}^{n})V_{p}+b\left(x_{i}^{n},t\right)$$

where ${\ddot{u}}_{i}^{n}$ is the acceleration at point $x_{i}$ in time step n, $V_{p}$ is the volume of the material point at p, and $b\left(x_{i}^{n},t\right)$ the body force applied to $x_{i}$ in time step n.

The time-stepping scheme adopts the Verlet-velocity difference scheme, and the acceleration of the material point is expressed as [26]

(15) $${\ddot{u}}_{i}^{n}=\frac{u_{i}^{n+1}-2u_{i}^{n}+u_{i}^{n-1}}{\Delta t^{2}}$$

According to Silling [26], the stability condition reads

(16) $$\Delta t\leq \sqrt{\frac{2\rho }{\sum_{j=1}^{N_{i}}C_{ij}V_{j}}}\;$$

where $C_{ij}=\left|C\left(x_{j}-x_{i}\right)\right|=\left|\partial f/\partial \eta \right|$.

In PD numerical calculation, the initial position and the initial velocity need to be provided in advance. The position, velocity, and acceleration of the material point will update in each time step, and finally the interaction force between material points will be calculated.

## 3. Numerical Modeling

### 3.1. Simplification of Gear Honing Process

**Figure 3:**
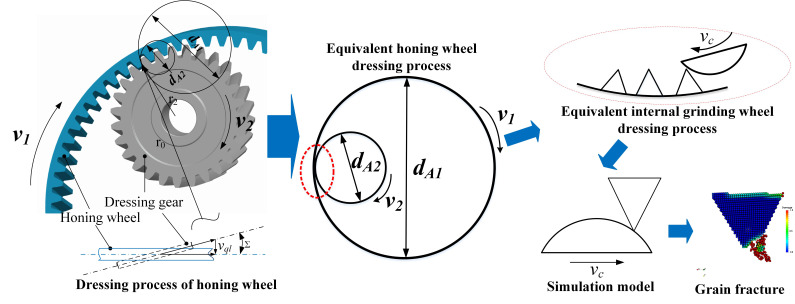
Simplification of gear honing process.

**Figure 4:**
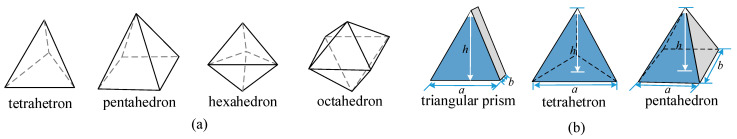
Shape of CBN grains, (**a**) in reference [31]; (**b**) current study.

**Figure 5:**
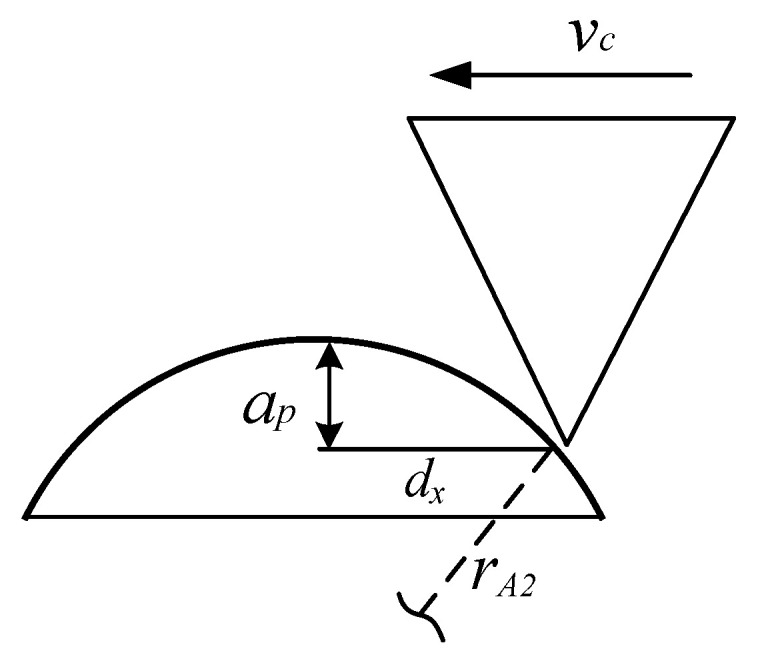
Simulation scheme.

In the honing wheel dressing process, the dressing gear and honing wheel remove materials from each other by contacting in the meshing area. This is a complex process that the CBN grain is fractured, while the honing wheel is worn. Numerical modeling of the real honing wheel dressing process will lead to a huge amount of computation and a waste of computing resources, so it is necessary to simplify the simulation process. According to T. Bergs [7], we simplified the honing wheel dressing process into an equivalent internal grinding wheel dressing process, as shown in Figure 3, where $d_{A1}$ is the analogous diameter of honing wheel and $d_{A2}$ is the analogous diameter of dressing gear. CBN grains are randomly distributed on the honing wheel. Due to the complex geometry of abrasive grains, their shapes usually vary between tetrahedral and octahedron [5], as shown in Figure 4a. In this paper, we set the shapes of abrasive grains to be triangular prism, tetrahedron and pentahedron, whose sizes are depicted as a, b, and h (Figure 4b). As the simulation scale is very small and the size of the honing wheel is usually much larger than the dressing gear, the motion of the abrasive grain on honing wheel can be approximately regarded as a linear movement relative to the dresser. Therefore, the analogous dressing process can simplified as Figure 5, in which $v_{c}$ is the dressing speed, $r_{A2}$ is the analogous radius of dressing gear, $a_{p}$ is the dressing depth, and $d_{x}$ is the horizontal distance that the grain moves and can be represented as $d_{x}=\sqrt{r_{A2}^{2}-{\left(r_{A2}-a_{p}\right)}^{2}}$.

### 3.2. Simplification of Gear Honing Process

For simplicity, the following hypothesis is adopted: (1) the physical and mechanical properties of the materials are isotropic; (2) the influence of temperature on grain fracture is ignored; (3) the dresser is assumed to be a rigid body, therefore the wear of the dresser during dressing process is neglected; (4) there are no initial micro-cracks in the CBN grains.

The simulations are implemented via the LAMMPS [32].

As shown in Figure 5, a dresser with the density of 3515 kg·m^3^ is clamped at the bottom. The analogous radius of dressing gear is taken as *r_A2_* = 0.5 mm. An isotropic grain, with the size of a, b, and h, moves in a straight line with the speed $v_{c}$. The dressing speeds of honing wheel are very slow compared with grinding and the value of $v_{c}$ lies between 0.5–15 m/s [7]. The horizontal distance $d_{x}$ and the dressing depth $a_{p}$ take different values in different simulations. The density ρ, Young’s modulus E and Poisson’s ratio v for CBN grain are 3480 kg·m^3^, 706 GPa, and 0.15, respectively. According to [33], the energy release rate $G_{c}\;$= 3 J·m^−2^. Therefore, in terms of Equations (9) and (11), we have c = 3.81 × 10^22^ GPa·m^−4^, κ = 1.32 × 10^22^ GPa·m^−4^, and $w_{c}\;$= 7.55 × 10^20^ J·m^−6^. A three-dimensional cubic grid with spacing Δ*x* = 5 μm is used. The horizon *δ* = 3Δ*x*, and time step Δ*t* = 1 × 10^−9^ s.

## 4. Results and Discussion

### 4.1. Model Validation

To validate the effectiveness of the current PD model, we carried out an equivalent experiment similar to the simulation scheme shown in Figure 5. The experiment results were compared with the simulation results, in which the dressing speed, grain geometry, and dressing direction were the same.

#### 4.1.1. Experimental Setup

**Figure 6:**
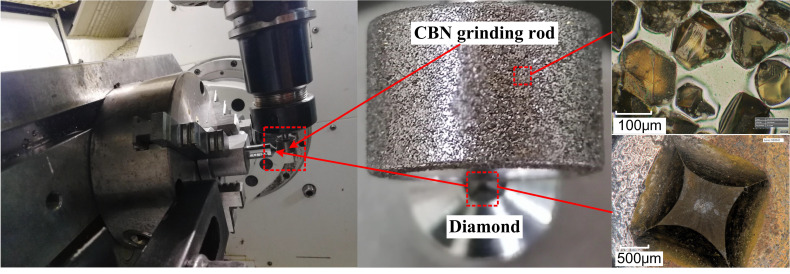
Experiment setup for of CBN grain based on simulation scheme.

As shown in Figure 6, a commercial CBN grinding rod (ceramic-based CBN grinding rod, Besdia Inc, China) with the grit size 100 was fixed to the toolholder of the Mazak 5-axis machining center, and a diamond dresser is fixed to the chuck of machining center. During the relative motion, the grinding rod moved linearly with the toolholder at a low speed of $v_{c}$ = 5 m/s without rotation, while the dresser remained stationary. The dressing depth was set as $a_{p}$ = 30 μm. The fracture topography was observed by Hirox-HRX01.

#### 4.1.2. Comparison of Simulation and Experimental Results

**Figure 7:**
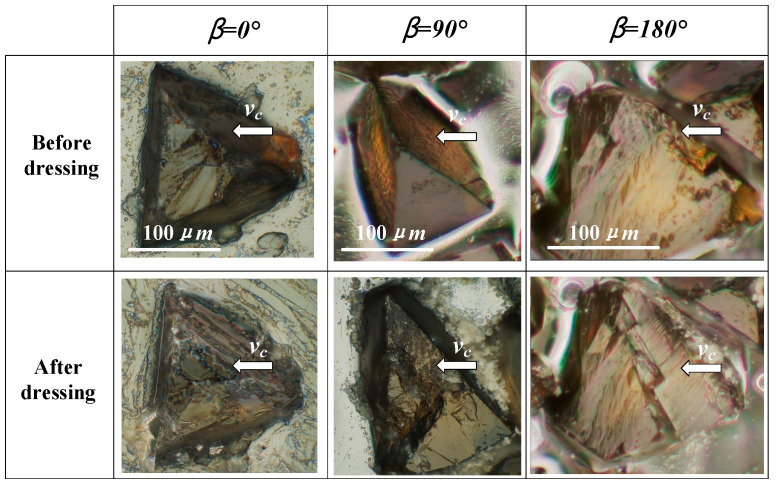
Experimental results for tetrahedron shaped CBN grains with different orientations before and after dressing.

**Figure 8:**
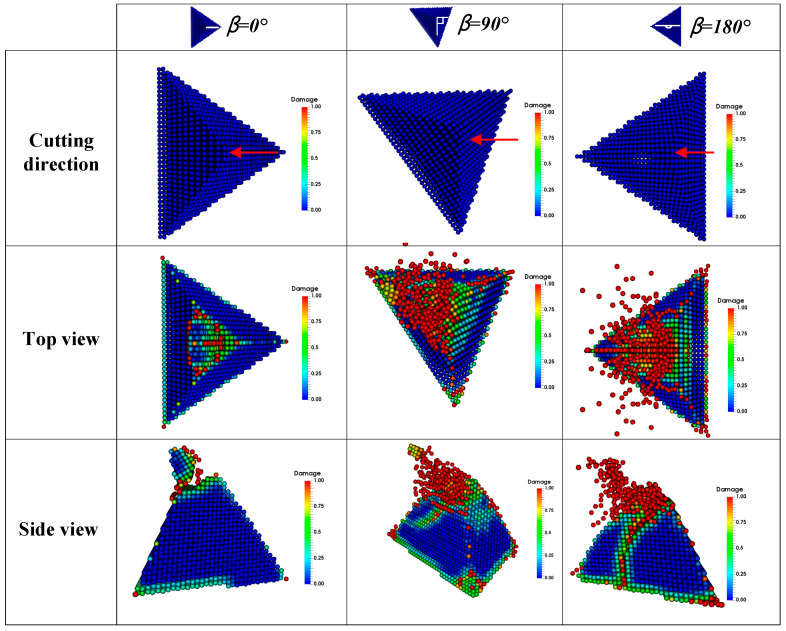
Simulation results for tetrahedron shaped abrasive grain with different orientations.

The topography of the abrasive grains in the contact area on the grinding rod is random. Therefore, in order to visually compare the simulation results with the experimental results, three kinds of tetrahedral shaped abrasive grains with different orientation were selected in advance during the experiment. The changes of their shapes were observed before and after dressing, and the sizes of the abrasive grains are shown in Figure 7, where β is the defined grain orientation angle, as depicted more precisely in Figure 8. Correspondingly, three different tetrahedral shaped grains with the dimensions of a = 100 μm and h = 84.7 μm were established in the simulation, as shown in Figure 8.

Figure 7 and Figure 8 show the experimental and simulation results before and after dressing respectively. When the grain orientation angle β = 0°, the fracture surface is almost flat and the fracture volume is relatively small, which is similar to the simulation result. When β = 90°, the grain has a steep fracture surface that nearly spreading all the way to the root of the grain, which is almost identical to the simulation result. When β = 180°, the abrasive grain also produces a steep fracture surface, which is nearly the same as the fracture surface at β = 90° from the experimental results and steeper than that at β = 90° from the simulation results, indicating that the simulation results are consistent with the fracture evolution trend revealed by the experimental results although they do not exactly match the experimental results. T. Bergs et al. [18] revealed a similar fracture pattern for CBN grains. Overall, the experimental results show that the model in this paper is capable of predicting the CBN abrasive fracture behavior reasonably.

### 4.2. Fracture Evolution of CBN Grain

**Figure 9:**
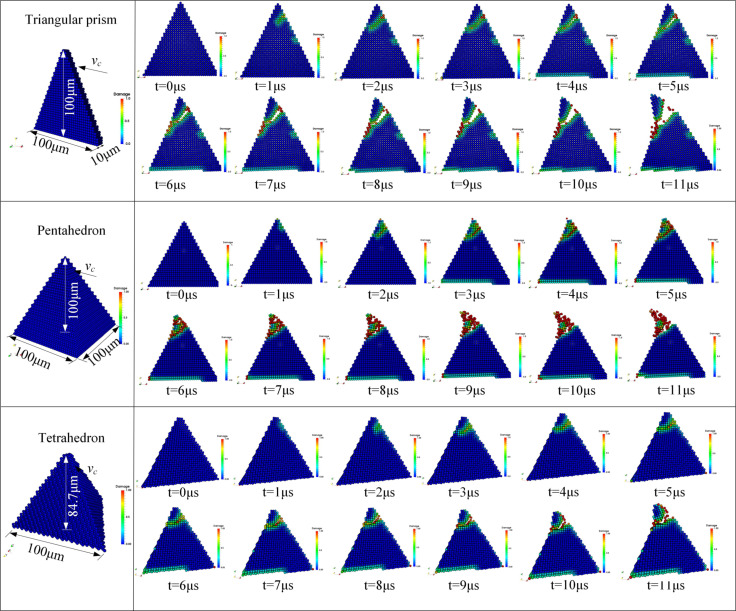
Crack initiation and propagation of different shaped CBN grains.

**Figure 10:**
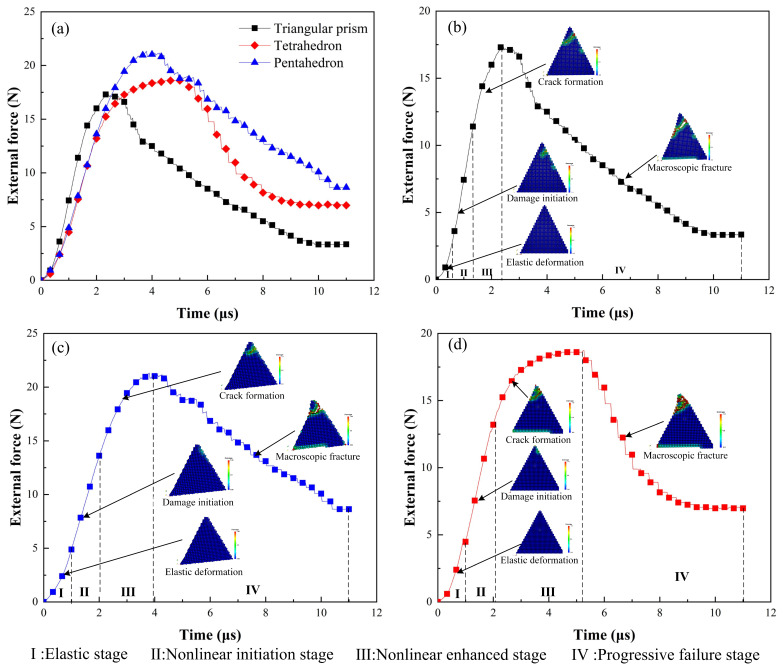
Comparison of force-time curves during the crack initiation and propagation process for three types of abrasive grains (**a**), description of crack initiation and propagation stages for tetrahedral grain (**b**), pentahedral grain (**c**), and triangular prism grain (**d**).

In this section, we will focus on the mechanism of crack initiation, propagation and fragmentation of CBN grains during dressing process, and reveal the main stages of fracture evolution. The topography of the abrasive grains are shown in Figure 4b, and the crack initiation and propagation patterns were compared for the three shaped abrasive grains. The dressing speed is set to $v_{c}$ = 5 m/s and the dressing depth $a_{p}$ = 30 μm. In order to quantitatively describe the fracture pattern, the external force applied to the abrasive grain by the diamond is recorded during the simulation. The sizes and the crack propagation states of the three abrasive grains at different time steps are shown in Figure 9, and the corresponding external forces change with loading time as shown in Figure 10a. The light-colored areas shown in Figure 9 represent the local damage factor lies in 0 < φ < 1 (Equation (13)), indicating that the bonds of material points in the local area are not broken and only damage is produced; the red areas denote φ = 1, indicating that the bonds of material points are broken and the abrasive grain produces local cracks or fractures.

It is observed that the crack initiation and expansion of the three types of abrasive grains have a similar pattern, i.e., the abrasive grains first experienced a linear elastic deformation, and the corresponding external force curve was linear; then, the abrasive grains produced local damage in the contact region, without forming cracks yet, and the external force changed from linear to weak non-linear; Subsequently, the local damage expanded into local cracks, and tiny cracks were formed in the contact area of the abrasive grains, and the corresponding external force first increased slowly, followed by a small oscillation; Finally, the local cracks developed further into macro fractures with obvious openings, followed by a rapid fragmentation. Correspondingly, the external force decreased rapidly until the end of the simulation.

Therefore, the whole process of crack initiation and expansion can be divided into four stages: elastic deformation, damage initiation, crack formation, and macro fracture. Likewise, the change of the external forces during the crack expansion can well manifest these four stages, which also underwent four stages: elastic stage, nonlinear emerging stage, nonlinear enhanced stage, and progressive failure stage. It is worth noting that the time of occurrence and duration of each deformation phase differ slightly for different shaped abrasive grains. This is mainly due to the dimensions of the three abrasive grains are not exactly the same, resulting in the curves of force and time for different abrasive grains are not fully consistent, namely, there is a size effect. The crack propagation has a similar pattern to the crack propagation of other brittle materials such as concrete [34] and Lac du Bonnet granite [35].

### 4.3. Stress Analysis of CBN Grain during Fracture Process

**Figure 11:**
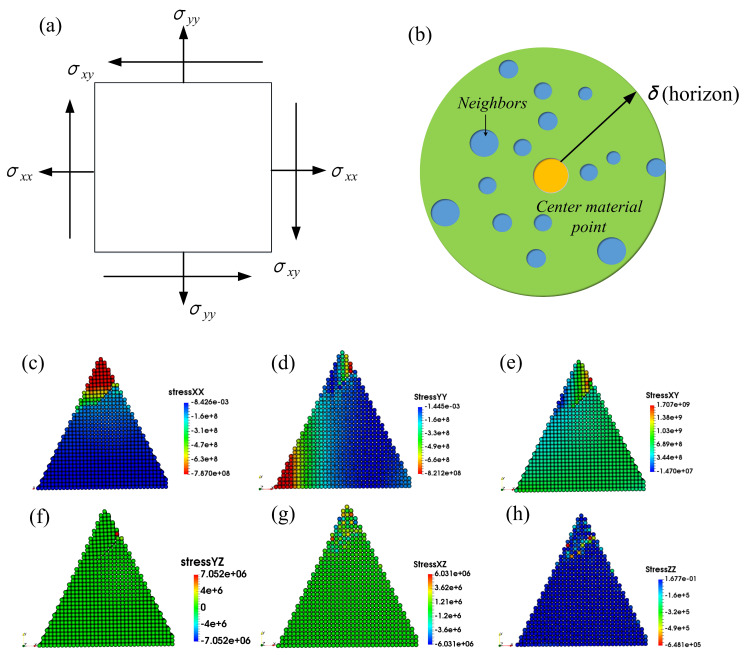
Schematic diagram of stress components (**a**), stress calculation scheme within PD horizon (**b**), and stress distribution of $\sigma_{xx}$, $\sigma_{yy}$, $\tau_{xy}$, $\tau_{xz}$, $\tau_{yz}$, and $\sigma_{zz}$ (**c**–**h**).

The forms of grain fracture and the several stages of fracture have been discussed above, but it is not yet certain how the various forms and stages of fracture arise. We know that the damage initiation and fracture evolution of a material is closely related to its stress. When the stress of a brittle material exceeds its strength limit, it begins to damage and fracture. Therefore, the cause of grain fracture can be described by studying the characteristics of stress. The stress applied to each material point of the abrasive grain during the dressing process are defined in Figure 11a. According to [36,37], the stress components at material point i can be expressed as $\sigma_{\alpha \beta }=\frac{1}{\Omega }\left(\underset{i}{\sum }m_{i}v_{i\alpha }⊗v_{i\beta }+\frac{1}{2}\underset{i\neq j}{\sum }r_{ij\beta }F_{ij\alpha }\right)$, where α,β=x,y,z are the Cartesian components, Ω is the volume of the domain within the PD horizon of material point i. $m_{i},\;v_{i\alpha }$ and $v_{i\beta }$ are the mass, the α component and β component the velocity of material point i, respectively. $r_{ij*}$ and $F_{ij*}=\int \int \left(cs\cdot n+\kappa \gamma \right)dV_{i}dV_{j}$ are the distance and interaction force components between material points within the horizon. As depicted in Figure 11b, the orange material point is the center of the local sphere with a horizon $\delta \;(\Omega =4{\pi \delta }^{3}/3)$, with other blue points in the same sphere and the subscript “I” and “j” refer to them. The stress tensor $\sigma_{\alpha \beta }$ is subjected to the center point, and the summation extend over all the material points in the horizon. $\sigma_{xx}$,${\;\sigma }_{yy}$ and $\sigma_{zz}$ are the normal stresses in the x, y and z directions, and $\sigma_{xy}$, $\sigma_{xz}$ and $\sigma_{yz}$ represent the shear stresses. Therefore, the stress distribution of the whole model would be achieved after the iteration over all the material points of the grain, as shown in Figure 11c–h.

**Figure 12:**
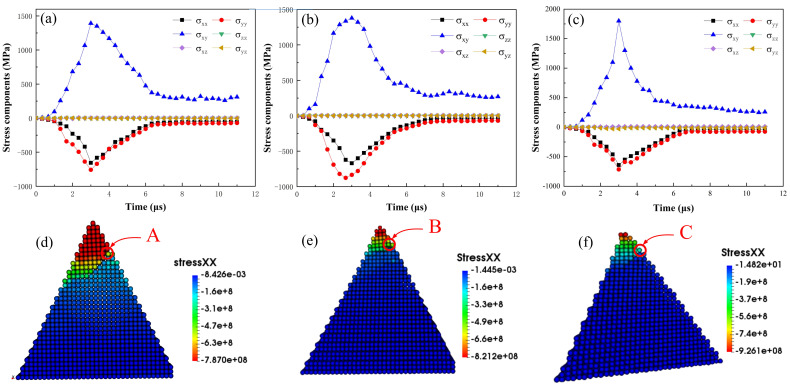
Stress components of material points A, B and C for different abrasive grains: (**a**) triangular prism, (**b**) pentahedron, (**c**) tetrahedron, and (**d**–**f**) show the locations of material points A, B and C.

To quantitatively describe the variation of stress components over time, a material point on the crack growth path of each of the three abrasive grains was selected respectively. The locations of the selected points A, B and C are shown in Figure 12d–f, and the variation of stress components are shown in Figure 12a–c. The stress components exhibited similar trends for the selected material points. During the simulation, the abrasive grain moved along the x-negative direction in the dressing process, and the models were symmetric about the xy plane. It is clear from Figure 11c–e and Figure 12d–f that the values of $\sigma_{xx}$, $\sigma_{yy}$ and $\sigma_{xy}$ in the crack formation region are much larger than those of $\sigma_{xz}$, $\sigma_{yz}$ and $\sigma_{zz}$, indicating that the damage initiation, propagation, cracking, and fracture of the abrasive grains are mainly contributed by $\sigma_{xx}$, $\sigma_{yy}$ and $\sigma_{xy}$. The normal stresses $\sigma_{xx}$ and $\sigma_{yy}$ are compressive stresses for different grains. This is due to the compressive pressure imposed by the dressing tool in the contact area [37]. Under high compressive stress, brittle materials tend to exhibit shear fracture [38,39]. Meanwhile, $\sigma_{xy}$ is high near the interface of crack initiation and propagation, and very low in other areas. When the shear stress is larger than material flow stress, the material dislocation emission will take place [19,33]. Hence, the formation and growth of fractures are mainly caused by normal stresses in the dressing direction and shear stresses in the symmetry plane. In addition, it is clear from Figure 11e and Figure 12 that the maximum shear stress is larger than the maximum normal stress, indicating that shear stress is one of the dominant factors leading to the fracture of the abrasive grains.

**Figure 13:**
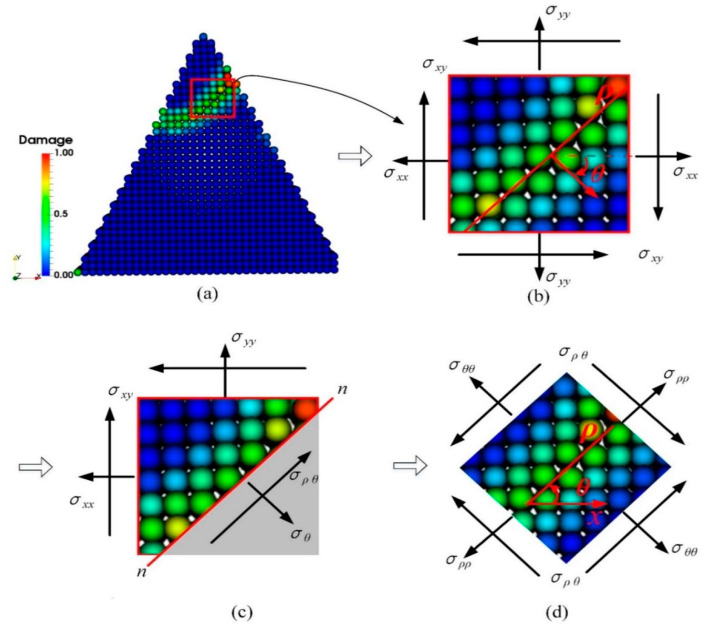
Stress state in the crack propagation path. (**a**) Damage pattern for triangular prism grain, (**b**) a micro-element taken from the crack propagation path, (**c**) stress analysis of section n-n, and (**d**) stress components in coordinate ρ−θ.

Although $\sigma_{xx}$ and $\sigma_{yy}$ are compressive stresses, it does not mean that compressive stress is a major factor leading to fracture of the abrasive grains. Thus, take a micro-element in the crack propagation path of the abrasive grain, and the stress state in orientation θ of this micro-element is shown in Figure 13. As shown in Figure 13a, a micro-element is taken in the crack propagation path of the triangular prism grain, and its stress analysis is shown in Figure 13b. Section the element in the crack propagation path by line n-n, then the normal stress and shear stress on the section can be represented as

(17) $$\sigma_{\theta \theta }=\frac{\sigma_{xx}+\sigma_{yy}}{2}+\frac{\sigma_{xx}-\sigma_{yy}}{2}cos2\theta +\sigma_{xy}sin2\theta$$

(18) $$\sigma_{\rho \theta }=\frac{\sigma_{xx}-\sigma_{yy}}{2}sin2\theta -\sigma_{xy}cos2\theta$$

**Figure 14:**
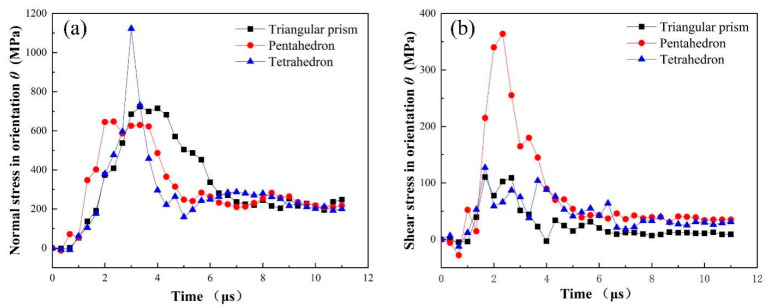
Normal stress (**a**) and shear stress (**b**) of material points A, B and C in orientation *θ*.

It can be seen from Figure 11 that the initial fracture propagation angle for different grains are nearly the same, i.e., $\theta \approx 45^{{}^{\circ}}$. Thus the normal stress and shear stress in orientation θ at point A, B and C can be depicted as Figure 14. It is evident that the normal stress in section n-n is positive, indicating that the abrasive grain is subjected to tensile stress in the crack expansion path, i.e., tensile stress may be one of the reasons for the cracking of the abrasive grain. To determine this, we further represent Figure 13c as Figure 13d by converting the stresses in the original x-y coordinate system to ρ−θ coordinate system. It is clear from Figure 13d that the cracks split under tensile stress $\sigma_{\theta \theta }$ and slip under shear stress $\sigma_{\rho \theta }$. Therefore, it can be concluded that the tensile and shear stresses in the section along the crack propagation path are responsible for the cracking and propagation of the abrasive cracks, which coincides with the conclusion obtained in References [36,40].

### 4.4. Fracture Mode Analysis

#### 4.4.1. Modes of Crack Propagation

**Figure 15:**
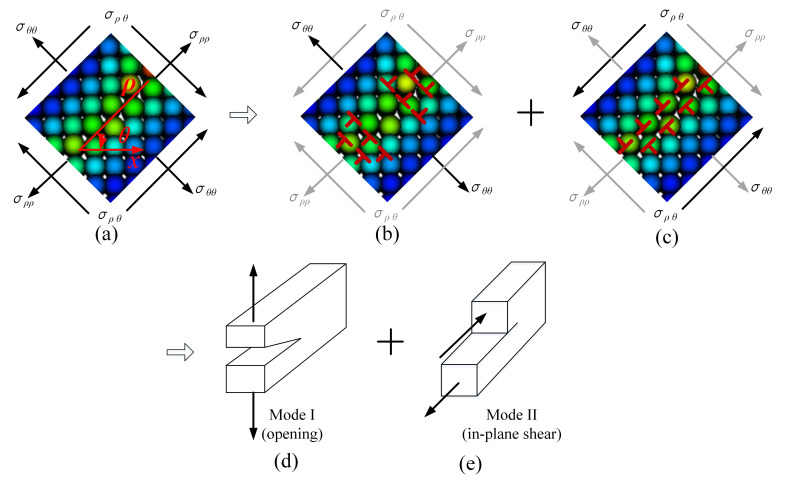
Modes of fracture propagation for CBN grain: (**a**) stress state in coordinate ρ−θ, (**b**) glide motion under tensile stress $\sigma_{\theta \theta }$, (**c**) glide motion under shear stress $\sigma_{\rho \theta }$, (**d**) opening mode (Mode I), and (**e**) in-plane shear (Mode II).

According to fracture mechanics, cracks can be classified into three types, namely, opening (mode I), sliding (mode II) and tearing (mode III). Wherein mode I corresponds to normal separation of the crack face under tensile stress; mode II corresponds to longitudinal relative shearing of the crack face occurring perpendicular to the direction of the crack front edge; and mode III corresponds to the lateral relative shearing parallel to the direction of the crack front edge between crack faces. It is clear from the previous discussion that CBN abrasive fracture is induced by a combination of normal and shear stresses in the cross section in the crack propagation path. Therefore, the crack propagation of CBN grain should contain both mode I cracks and mode II (or mode III) cracks. For further confirmation, we decompose the stress in direction *θ* of the crack expansion path, as shown in Figure 15. Figure 15b shows the dislocation diagram of the crack under tensile stress. The crack is opened under the action of $\sigma_{\theta \theta }$, which indicates that there is an edge-type dislocation distributed along the crack plane, and the dislocation climbs under the action of the normal stress, leading to the crack propagation. Figure 15c shows the dislocation diagram under shear stress. The crack slides away under the action of $\sigma_{\rho \theta }$, and similarly, the dislocation slips under the shear stress, leading to crack propagation. Comparing the normal stress and shear stress shown in Figure 14, it is clear that the normal stress is much larger than the shear stress. Meanwhile, the tensile strength is smaller than the shear strength [38,39], so the cracks will first occur under the tensile stress; when the magnitude of the shear stress exceeds the shear strength, shear sliding occurs. Therefore, it can be determined that the fracture of CBN grains consists of Mode I and Mode II, where the former is dominant over the latter [37,41]. The intrinsic mechanism is that a brittle crack always tends to find a direction that minimizes the shear load in which propagation occurs, which is consistent with the crack propagation process caused by the breakage of a material bond crossing the crack face subjected to tensile loading [33].

#### 4.4.2. Instability of Crack Propagation Path

**Figure 16:**
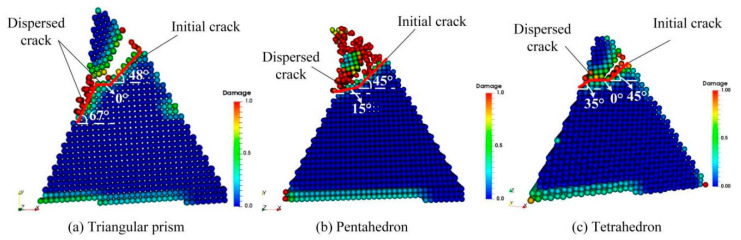
Crack propagation paths for different abrasive grains. (**a**) Triangular prism, (**b**) Pentahedron, and (**c**) Tetrahedron.

In the above discussion about the CBN abrasive crack propagation modes, it is tacitly assumed that the crack has been propagating along the original path and that the propagation process is more inclined to be subjected to the tensile stress component under external load, which provides an intuitive basis for revealing the simple crack opening path. However, the fact is that crack propagation can also deviate from its initial path. Figure 16 shows the crack propagation paths for three different abrasive grains. It can be clearly observed that there is obvious branching phenomenon during the crack propagation of different shaped abrasive grains, and the branching paths are different. For example, the Triangular prism grain initially cracked at an angle of $48^{{}^{\circ}}$ to the horizontal, then expanded at $0^{{}^{\circ}}$, and finally spread and debonded at $67^{{}^{\circ}}$; the Pentahedron grain initially cracked at $45^{{}^{\circ}}$, then spread and debonded at $15^{{}^{\circ}}$; the Tetrahedron grain initially cracked at $45^{{}^{\circ}}$, then expanded along $0^{{}^{\circ}}$ direction, and finally developed and shed along $35^{{}^{\circ}}$. This indicates that the initial crack propagation paths for different abrasive grains are almost the same, but the propagation paths after diverging vary depending on the shape of the abrasive grains and do not have the same regularity as the initial crack propagation. Therefore, the crack propagation path after diverging shows an unstable characteristic.

**Figure 17:**
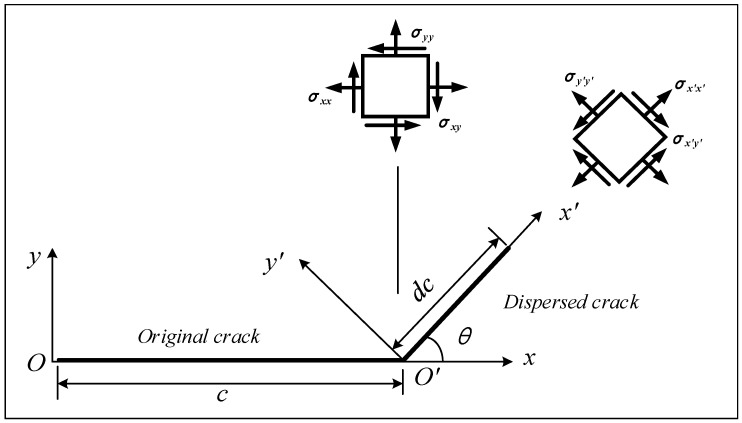
Unstable crack propagation model for CBN grain.

In order to further illustrate the instability of crack propagation path, we construct a crack propagation model in the plane, as shown in Figure 17. Suppose that there is a crack with length c in the plane, and let the propagation of this crack diverge, and the incremental crack dc after the divergence is at an angle *θ* from the initial crack. Assuming that the propagation direction of incremental crack is always along the direction that brings about the fastest reduction in the total energy of the system. Therefore, for an isotropic material, the problem translates into finding the value of *θ* that maximizes the mechanical energy release rate G.

Here we discuss in two cases, the first case is that the crack is under pure mode I loading, and the second is a mixture of mode II and mode I loading.

For pure mode I loading, the initial stress component at the crack tip can be expressed as [35]

(19) $$\left\{\begin{array}{c} \sigma_{xx}\\ \sigma_{yy}\\ \sigma_{xy} \end{array}\right\}=\frac{K_{I}}{{\left(2\pi r\right)}^{1/2}}\left\{\begin{array}{c} cos\left(\theta /2\right)\left[1-sin\left(\theta /2\right)sin\left(3\theta /2\right)\right]\\ cos\left(\theta /2\right)\left[1+sin\left(\theta /2\right)sin\left(3\theta /2\right)\right]\\ sin\left(\theta /2\right)cos\left(\theta /2\right)cos\left(3\theta /2\right) \end{array}\right\}$$

where$\;K_{I}$ is the stress intensity factor.

If the crack propagates along direction *θ*, then the normal and shear stress components in the new coordinate system can be obtained by coordinate transformation

(20) $$\left\{\begin{array}{c} \sigma_{x^{\prime }x^{\prime }}\\ \sigma_{y^{\prime }y^{\prime }}\\ \sigma_{x^{\prime }y^{\prime }} \end{array}\right\}=\frac{K_{I}}{{\left(2\pi r\right)}^{1/2}}\left\{\begin{array}{c} cos\left(\theta /2\right)\left[1+sin^{2}\left(\theta /2\right)\right]\\ cos^{3}\left(\theta /2\right)\\ sin\left(\theta /2\right)cos^{2}\left(\theta /2\right) \end{array}\right\}$$

The stress formulas in (19) and (20) can be simplified as

(21) $$\sigma_{ij}=K_{I}{\left(2\pi r\right)}^{-1/2}f_{ij}^{I}$$

(22) $$\sigma_{i^{\prime }j^{\prime }}=K_{I}{\left(2\pi r\right)}^{-1/2}f_{i^{\prime }j^{\prime }}^{I}$$

Let

(23) $${K^{\prime }}_{I}\left(\theta \right)=K_{I}f_{y^{\prime }y^{\prime }}^{I},\;{K^{\prime }}_{II}\left(\theta \right)=K_{I}f_{x^{\prime }y^{\prime }}^{I},\;{K^{\prime }}_{III}\left(\theta \right)=0$$

where ${K^{\prime }}_{i}\left(\theta \right)$ is the transformed stress intensity factor, which determines the crack field after deviation of the crack direction. Thus, the variation of mechanical energy release rate with angle θ can be expressed as

(24) $$G\left(\theta \right)={K^{\prime }}_{I}^{2}\left(1-v^{2}\right)/E+{K^{\prime }}_{II}^{2}\left(\theta \right)\left(1-v^{2}\right)/E$$

where ν and E are the Poisson’s ratio and Young’s modulus, respectively.

**Figure 18:**
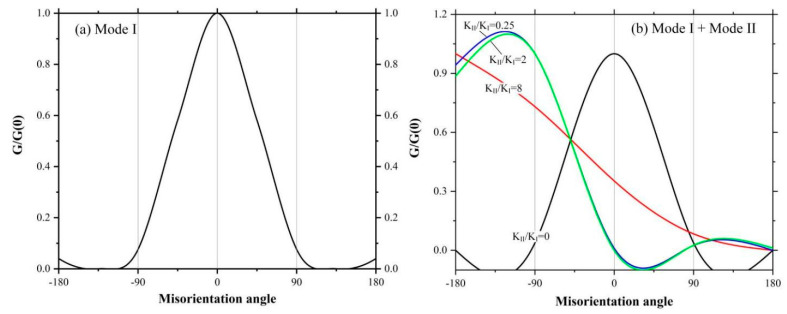
The variation of normalized mechanical energy release rate with crack misorientation angle *θ*: (**a**) pure mode I loading; (**b**) mixed loading of mode I and mode II.

The function $G\left(\theta \right)$ is normalized by the value of $G\left(0\right)$ expanded along its own plane and then plotted in Figure 18a. It shows that $G\left(\theta \right)$ takes the maximum value when θ = 0, which means that the crack always tends to propagate along its own direction, i.e., the propagation of mode I crack has ‘path stability’.

For the combined loading of mode I and II, the analysis process is the same as above, except that the contribution of the crack field of mode II needs to be considered for solving the stress component, and the corresponding transformed stress intensity factor is expressed as

(25) $${K^{\prime }}_{I}\left(\theta \right)=K_{I}f_{y^{\prime }y^{\prime }}^{I}+K_{II}f_{y^{\prime }y^{\prime }}^{II},\;{K^{\prime }}_{II}\left(\theta \right)=K_{I}f_{x^{\prime }y^{\prime }}^{I}+K_{II}f_{x^{\prime }y^{\prime }}^{II},\;{K^{\prime }}_{III}\left(\theta \right)=0$$

where the coordinate transformation matrix for mode II cracks is [35]

(26) $$f_{i^{\prime }j^{\prime }}^{II}=\left\{\begin{array}{c} sin\left(\theta /2\right)\left[1-3sin^{2}\left(\theta /2\right)\right]\\ -3sin\left(\theta /2\right)cos^{2}\left(\theta /2\right)\\ cos\left(\theta /2\right)\left[1-3sin^{2}\left(\theta /2\right)\right] \end{array}\right\}$$

Similarly, the mechanical energy release rate at mixed loading can be derived from Equation (24), which is normalized as shown in Figure 18b. It shows that the normalized mechanical energy release rate varies with the crack misorientation angle as the ratio of mode II to mode I crack stress intensity factor varies. Moreover, due to the action of shear force, the crack propagation direction gradually deviates from the original path, i.e., it has “instability of propagation direction”. Therefore, the crack branching phenomenon during the CBN grain dressing process described above is in fact mainly contributed by shear stress, which makes the crack propagation path unstable.

## 5. Conclusions

The internal honing wheel dressing process is simplified as an internal grinding wheel dressing process, and the bond-based Peridynamic simulations are conducted to investigate the fracture mechanism of CBN grain in the dressing process. Based on the discussions above, the conclusions can be drawn as follows:

(1) The bond-based Peridynamic method considering the bond rotation effect is proven suitable for simulating the fracture mechanism of single CBN grain.

(2) In the dressing process, the fracture evolution of CBN grains mainly undergo four stages: elastic deformation, damage initiation, crack formation, and macro fracture. Correspondingly, the force-time curves for different grains also go through four stages: elastic stage, nonlinear emerging stage, nonlinear enhanced stage, and progressive failure stage. The fracture propagation process shows high similarity with the fracture patterns of other brittle materials.

(3) The crack initiation and propagation of CBN grain are mainly determined by the tensile and shear stress of material points. When the tensile stress of a material point exceeds the tensile strength limit, the bond that connect neighbored material points will break along the axial direction and the grain will produce mode I fracture. When the shear stress of a material point surpasses its shear strength, the bond will break in the tangential direction and the grain will form mode II fracture. During the fracture evolution process of CBN grain in dressing, mode I fracture is dominant over mode II.

(4) There are stable and unstable stages in the process of fracture evolution for different CBN grains. In the stable stage, the cracks for different grains propagate along the direction about 45° from horizontal, which is mainly a result of the tensile stress perpendicular to the crack growth direction. In the unstable stage, the fracture propagation paths are unstable and will deviate from their original growth paths, which is mainly due to the action of shear stress and displays different patterns for different grains.

(5) The CBN grains and the dressing process of honing wheel are simplified in the modeling of this paper. The results are consistent with the experimental results and related studies, which indicate that the model and simulation method of this paper are reasonable and have a reference value for conducting similar simulation studies.

## Data Availability

Not applicable.

## Abbreviations

CBN	cubic boron nitride
PD	Peridynamics
MD	mechanical dressing
EDD	electrical discharge dressing
ELID	electrolytic in-process dressing
PLD	pulsed laser dressing
FEM	the finite element method
SPH	smooth particle hydrodynamics
PMB	prototype micro elastic brittle
a, b, and h	simplify the size of abrasive grain
$a_{p}$	the dressing depth
$b\left(x,t\right)$	external body force
c	the material bond constant
$d_{x}$	the horizontal distance that the grain moves
E	the Young’s modulus
$F_{ij*}$	the interaction force components between material points within the horizon
$f\left(x,x^{\prime },t\right)$	PD bond force between material points x and *x*′
$G_{c}$	the critical energy of fracture
K	the bulk modulus of the material
$K_{I}$	the stress intensity factor
$K{\text{′}}_{i}\left(\theta \right)$	the transformed stress intensity factor
κ	the stiffness in the tangential direction
$m_{i}$	the mass
n	the direction vector of material bond
n	time step
$r_{A2}$	the analogous radius of dressing gear
$r_{ij*}$	the distance between material points within the horizon
s	the bond stretch ratio
t	time
$u\left(x,t\right)$	the displacement field
${\ddot{u}}_{i}^{n}$	the acceleration at point $x_{i}$ in time step n
$V_{p}$	the volume of the material point at p
v	the Poisson’s ratio
$v_{c}$	the dressing speed
$v_{i\alpha }$,$v_{i\beta }$	the α component and *β* component the velocity of material point *i*
$w_{c}$	the critical micro-potential energy density
$w_{\xi }$	the material micro-potential energy density
$\rho \left(x\right)$	mass density
η	the relative displacement vector between material points
ξ	the relative position vector between material points
β	the grain orientation angle
Ω	the volume of the domain within the PD horizon of material point *i*
$\sigma_{xx}$,${\;\sigma }_{yy}$ and $\sigma_{zz}$	the normal stresses in the *x*, *y* and *z* directions
$\sigma_{xy}$, $\sigma_{xz}$ and $\sigma_{yz}$	the shear stresses
